# Supplementary material for: The impact of consumer preferences on the evolution of competition in China’s automobile market under the Dual Credit Policy—A density game based perspective
Source: PLoS One. 2024 Mar 7;19(3):e0295947. doi: 10.1371/journal.pone.0295947 (PMC10919624; doi:10.1371/journal.pone.0295947)
Supplement: S1 File — (DOCX) [file pone.0295947.s001.docx]

Dataset

Table 1 Conventional Fuel Vehicle Market Sales, 1996-2018

| Time | Conventional Fuel Vehicle Market Sales（vehicles） |
| --- | --- |
| 1996 | 772914 |
| 1997 | 900020 |
| 1998 | 945003 |
| 1999 | 1081701 |
| 2000 | 1313725 |
| 2001 | 1545232 |
| 2002 | 2171445 |
| 2003 | 3179395 |
| 2004 | 3545153 |
| 2005 | 4240253 |
| 2006 | 5464867 |
| 2007 | 6647018 |
| 2008 | 7096202 |
| 2009 | 10315044 |
| 2010 | 13739649 |
| 2011 | 14477799 |
| 2012 | 15455000 |
| 2013 | 17890669 |
| 2014 | 19616969 |
| 2015 | 20884851 |
| 2016 | 23870667 |
| 2017 | 24021803 |
| 2018 | 22397359 |
