# Supplementary material for: The impact of consumer preferences on the evolution of competition in China’s automobile market under the Dual Credit Policy—A density game based perspective
Source: PLoS One. 2024 Mar 7;19(3):e0295947. doi: 10.1371/journal.pone.0295947 (PMC10919624; doi:10.1371/journal.pone.0295947)
Supplement: S3 File — (DOCX) [file pone.0295947.s003.docx]

**Table 4. Stability solutins for parameter changes in the game model**

| **Equilibrium Point** | **Parameter Constraint 1：**  $\boldsymbol{H}_{\boldsymbol{4}}\boldsymbol{>}\boldsymbol{H}_{\boldsymbol{2}}$ | | | **Parameter Constraint 2：** $H_{1}-H_{3}-H_{2}+H_{4}<0$  | | |  |
| --- | --- | --- | --- | --- | --- | --- | --- |
|  | $\boldsymbol{Det} \boldsymbol{J}_{\mathbf{1}}$ | **T**$\boldsymbol{r}\boldsymbol{J}_{\mathbf{1}}$ | **Stability** | $\boldsymbol{Det} \boldsymbol{J}_{\mathbf{1}}$ | **T**$\boldsymbol{r}\boldsymbol{J}_{\mathbf{1}}$ | **Stability** | |
| O（0，0） | $\boldsymbol{+}$ | $\boldsymbol{-}$ | ESS | $+$ | N | Instability | |
| $A_{1}$（0，1） | $\boldsymbol{-}$ | $\boldsymbol{-}$ | Instability | $+$ | $-$ | ESS | |
| $B_{1}$（1，0） | $\boldsymbol{-}$ | $\boldsymbol{-}$ | Instability | $+$ | $-$ | ESS | |
| $C_{1}$（1，1） | $\boldsymbol{+}$ | N | Saddle Point | $+$ | N | Saddle Point | |
| $D_{1}(\frac{H_{4}-H_{2}}{H_{1}-H_{2}-H_{3}+H_{4}},\frac{H_{4}-H_{2}}{H_{1}-H_{2}-H_{3}+H_{4}})$ | — | — | — | — | — | — | |
| **Evolutionary phase diagram** | 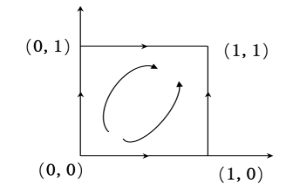 | | | 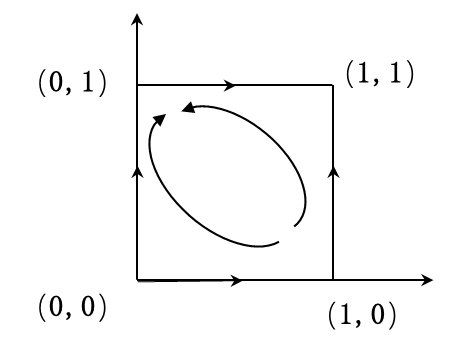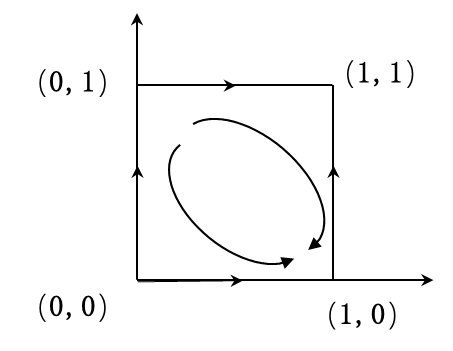 | | | |
| **Equilibrium Point** | **Parameter Constraint 3：**  $\boldsymbol{H}_{\boldsymbol{1}}\boldsymbol{>}\boldsymbol{H}_{\boldsymbol{3}}$ | | | | | | |
|  | $\boldsymbol{Det} \boldsymbol{J}_{\mathbf{1}}$ | | **T**$\boldsymbol{r}\boldsymbol{J}_{\mathbf{1}}$ | | **Stability** | | |
| O（0，0） | $\boldsymbol{+}$ | | N | | Saddle Point | | |
| $A_{1}$（0，1） | $\boldsymbol{+}$ | | $\boldsymbol{+}$ | | Instability | | |
| $B_{1}$（1，0） | $\boldsymbol{-}$ | | N | | Saddle Point | | |
| $C_{1}$（1，1） | $\boldsymbol{+}$ | | $\boldsymbol{-}$ | | ESS | | |
| $D_{1}\left( -\frac{H_{2}-R}{H_{1}-H_{3}}，-\frac{H_{2}-R}{H_{1}-H_{3}} \right)$ | — | | — | | — | | |
| **Evolutionary phase diagram** | 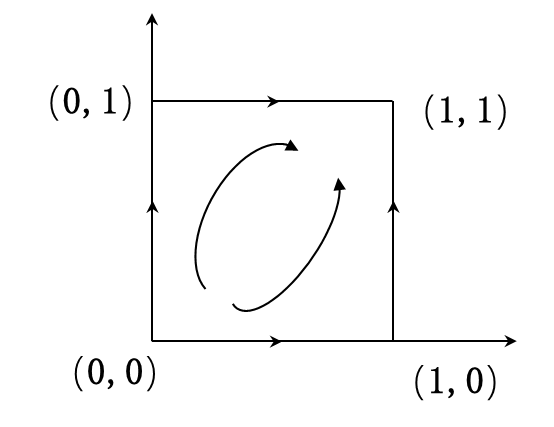 | | | | | | |

Note: "+" indicates greater than 0, "-" indicates less than 0, and "N" indicates uncertainty.
